# Minimally invasive aortic valve replacement with central cannulation: A cost-benefit analysis in a developing country

**DOI:** 10.1186/s43057-020-00019-y

**Published:** 2020-03-06

**Authors:** Mohammed Sanad, Hatem Beshir

**Affiliations:** 1 Department of Cardiothoracic Surgery, Faculty of Medicine, Mansoura University Hospitals, D17, F5. 60, El Gomhoria Street, Qism 2, Mansoura, Dakahlia 35516 Egypt; 2 Department of Cardiothoracic Surgery, Egypt Ministry of Health and Population, Nasser Institute for Research and Treatment, Cairo, Egypt; 3 Department of Cardiothoracic Surgery, Egypt Ministry of Health and Population, Alexandria Directorate, Alexandria, Egypt

**Keywords:** Aortic valve replacement, Minimally invasive cardiac surgery, Mini-sternotomy, Central cannulation

## Abstract

**Background:**

Minimally-invasive approaches to aortic valve replacement (MIAVR) are technically and logistically demanding. However, few centers have started using these approaches with standard equipment because of the limited resources. We sought to report intra- and postoperative clinical outcomes and address health resource utilization after MIAVR.

**Results:**

A total of 102 eligible patients who had aortic valve replacement were enrolled in a prospective, multicenter cohort study conducted from June 2015 to December 2017. Fifty patients underwent aortic valve surgery via upper inverted T-shaped hemi-sternotomy (MS), and 52 patients were operated using full sternotomy (FS) in two centers in a developing country. Central cannulation was performed in all cases. Major adverse cardiac events, pain, and wound complications were compared. A cost analysis was performed, and exposure and feasibility for cannulation were assessed. The mean length of MS skin incision was 5.82 ± 0.67 cm. Cumulative cross-clamp time was insignificant between both groups (91.87 ± 34.41 versus 94.91 ± 33.96 min; *p* = 0.66). MS exhibited shorter ventilation time (6.18 ± 1.86 versus 10.68 ± 12.78 h; *p* = 0.029) and intensive care stays (33.27 ± 19.75 versus 49.42 ± 47.1 h; *p* = 0.037). Major adverse cardiac events (MACEs) were compared, and MS group exhibited fewer transfusions (1.18 ± 0.89 versus 1.7 ± 0.97 units; *p* = 0.002), fewer pulmonary complications (1 (2%) versus 2 (3.8%); *p* < 0.001), and less sternotomy wound infection (1 (2%) versus 5 (9.6%); *p* = 0.048). Total operative mortality of 4.46% was recorded (*n* = 5). Significant cost reduction was recorded favoring MS; central cannulation saved $907.16 and carried a total cost reduction of $580 (9.3%) when compared with the FS approach (*p* < 0.0001).

**Conclusions:**

With a lack of logistics in developing countries, MIAVR not only has a cosmetic advantage but carries a significant reduction in blood use, respiratory complications, pain, and cost. MIAVR can be feasible, with a rapid learning curve in developing centers.

## Background

In developing countries, rheumatic heart disease remains the leading cause of aortic valve disease, and surgical aortic valve replacement (SAVR) through full sternotomy is the standard approach [1].

Several minimally invasive techniques for cardiac surgery had been presented to our country since 2011 to lessen the intrusiveness of the surgical procedures, reduce pain, and shorten hospital stays while sustaining equivalent quality and safety of SAVR with a better quality of life and few complications as compared with conventional SAVR while maintaining cost-effectiveness.

Multiple impediments face cardiac surgery in developing nations. The lack of subsidy, high expenses linked to surgical equipment, and lengthier training curve make minimally invasive SAVR difficult as a standard of care and place a burden on the healthcare systems.

Multiple techniques for minimal invasive SAVR were proposed to decrease the length of skin incision and sternal involvement. Upper J-shaped sternotomy, inverted T-shaped mini-sternotomy, C-shaped sternotomy, and right anterior thoracotomy (RAT) approaches were described [2].

Two strategies of arterial cannulation were proposed: the retrograde route with femoral arterial cannulation and the antegrade route via direct ascending aorta cannulation. Cannulation of the groin was designated for refining exposure during surgery, but irrespective of the higher cost, in some instances, this tactic may lead to infection, lymphoid fistula, arterial wall dissection, distal limb ischemia, and increased risk of neurological complications [3].

The aim of this study was to compare the surgical outcomes and cost-effectiveness of SAVR with central cannulation using mini-sternotomy (MS) to full sternotomy in a developing country without additional equipment.

## Methods

This prospective multicenter cohort study was conducted between June 2015 and December 2017 and included 112 adult patients who underwent surgical aortic valve replacement in 2 institutions. We followed the declaration of Helsinki regarding studies on human subjects [4].

The patients were operated by two surgical teams who performed surgery in both centers. Both surgical teams were oriented with conventional and minimally invasive surgical approaches.

There were 102 eligible cases who underwent isolated aortic valve replacement. Cases with valve calcifications and chronic endocarditis were included. Re-operative cases, emergency operation, patients with thoracic deformities, low ejection fraction (EF) (less than 40%), active infective endocarditis, porcelain aorta, and EuroSCORE II predicted mortality more than 10%, and those who had concomitant cardiac surgery were excluded to create two equal groups. Two years of follow-up were conducted, and the vital status was reported.

Twelve patients were excluded from our sample; two cases were excluded from the MS group for concomitant ascending aorta replacement, and ten cases were excluded from the FS group; three had re-operative SAVR, and 7 cases had a concomitant ascending aorta replacement.

The MS group included 50 patients, and the FS group had 52 patients. We analyzed the operative, short-term hospital outcomes, mid-term outcomes, and cost-effectiveness analysis of each technique.

### Preoperative stratification

Computerized tomography (CT) was performed preoperatively to all cases to assess the aortic root position, the alpha angle, and the sternal to root distance. Candidate patients for the mini-sternotomy approach required more than 50% of the aortic root behind the sternum [2]. CT is of crucial importance for the exclusion of porcelain aorta and has a better role in stratification of cases eligible for the right anterior mini-thoracotomy approach.

The patients were stratified into two groups according to their definitive surgical route based on surgeons’ preference:

1. *Mini-sternotomy (MS)* included 50 patients in whom SAVR was done via upper inverted T-shaped hemi-sternotomy.
2. *Full sternotomy (FS)* included 52 patients with conventional SAVR using full median sternotomy.

### Anesthetic protocol

Under full invasive monitoring, the anesthetic protocol encompassed induction with fentanyl (1–10 μg/kg), atracurium besylate (0.4–0.5 mg/kg) for muscle relaxation, and propofol infusion (100–300 μg/kg/h) for the maintenance of anesthesia to endorse early extubation. Pain medication included pethidine hydrochloride in the intensive care unit (ICU) and oral acetaminophen (500 mg) while in the ward.

### Surgical technique

Following general anesthesia, the FS group had a full sternotomy, while the MS group underwent a 5–7 cm upper sternotomy skin incision followed by a 10 cm upper inverted T hemi-sternotomy using a primary sternal blade for longitudinal osteotomy and a redo blade for the transverse sternotomy [2] at the level of the third intercostal space. The conventional group had a classical full median sternotomy.

**Fig. 1:**
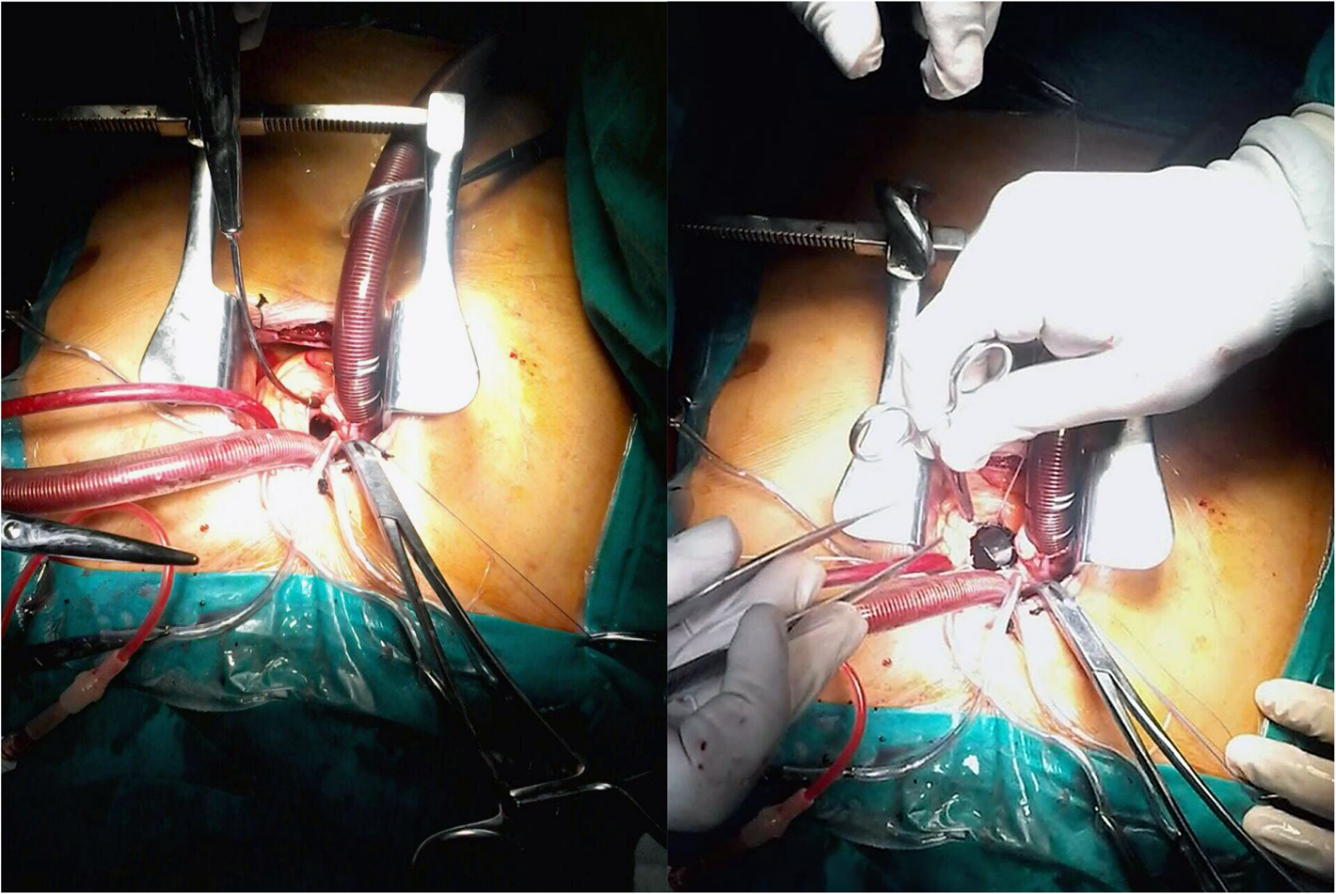
Intraoperative view of the surgical field with aortic cross-clamp, central cannulation, pulmonary artery vent, and full exposure to the aortic valve with the view of the valve prosthesis in place (right)

The pericardium was retracted to expose the aorta until the origin of the innominate artery. This approach allows the ascending aorta to come forward to obtain better exposure. Two concentric 2-0 polypropylene purse-string sutures were taken at the anterolateral aspect of the ascending aorta as high as possible (Fig. 1).

The cardioplegia cannula was inserted to maintain the least distance of 1 cm between the cross-clamp and the aortic cannulation site.

Cardiopulmonary bypass was instituted after systemic heparinization using central aortic and dual-stage venous cannulation in all cases. Induced systemic arterial hypotension < 90 mmHg was required during cannulation.

Myocardial protection was established using a standard central aortic cross-clamp followed by antegrade aortic root or direct coronary cold cardioplegia with moderate systemic hypothermia 28–32 °C. Venting was performed via catheterizing the right superior pulmonary vein or the pulmonary artery. The rest of SAVR was completed as the standard of care after evaluation of the valve pathology using an appropriately sized bioprosthesis or mechanical aortic valve prosthesis.

**Fig. 2:**
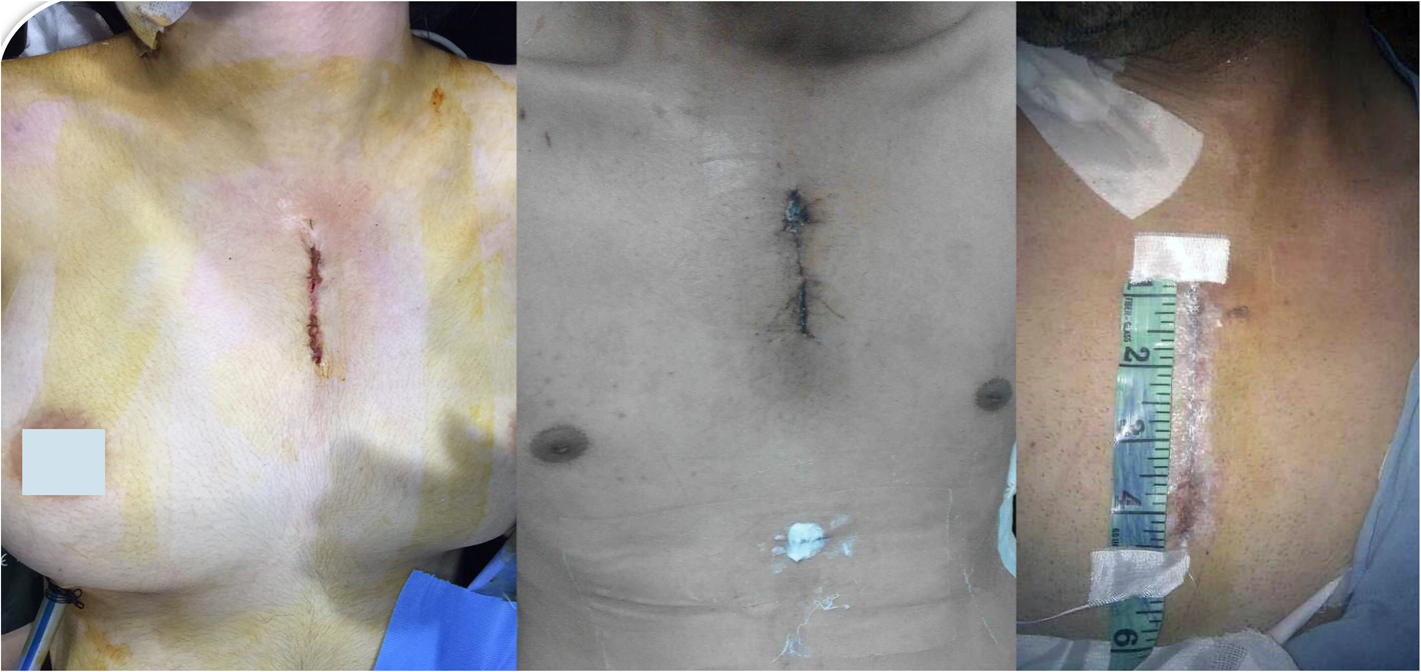
The incision for minimally invasive aortic valve replacement

Before weaning off bypass, a single subxiphoid chest drain was used from a 1 cm transverse skin incision and inserted through the pericardial cavity. In the case of pleural collection, a separate intercostal drain was inserted if needed (Fig. 2).

Following hemostasis, 4 to 6 stainless steel wires were used for closure of FS, while only 2 wires were used in the MS group.

### Study endpoints

The primary endpoint was postoperative mechanical ventilation (MV) and intensive care stay times (in hours). The secondary endpoints were cross-clamp (CCT), cumulative cardiopulmonary bypass time (CBP), pain score after 12 h (1–10), all-cause mortality, need for transfusion, cost analysis, and major adverse cardiac events (MACE) [5], which is a composite endpoint including cardiac death, myocardial infarction, secondary intervention/reoperation, ventricular arrhythmia, renal failure or multi-organ failure, and outcome after 2 years of follow-up. Total cost breakdown was logged for all patients conferring the institutions’ financial catalogs.

### Statistical analysis

Based on a pilot study of 10 patients of each technique, the mean hours of ICU stays were found to be 35.7 ±16.5 h in MS versus 45.87 ± 36.78 h in FS. The least significant total sample size of 94 patients was calculated to have a study power of 85% with an alpha error of 5%, a beta error of 15%, and an effect size of (Hedges’ g = (45.87 − 35.7)/29.520274 = 0.344509).

A total of 112 patients were enrolled, of which 102 patients met eligibility criteria. The data were tabulated and analyzed using the IBM SPSS software version 22.0 (IBM Inc., Chicago, IL, USA). Qualitative data were described using numbers and percents. Continuous variables were assessed for normality; normal variables were reported as mean ± standard deviation (SD), while non-normal variables were reported as the median and interquartile range (IQR).

A comparison between categorical variables was performed using the chi-square test (χ2-test). When more than 20% of the cells have expected count less than 5, correction for chi-square was conducted using Fisher’s exact test or Monte Carlo correction. Confidence intervals (95% CI) were calculated. For normally distributed data, a comparison between two independent populations was made using the independent *t* test. For abnormally distributed data, we used the Mann-Whitney *Z* test and Wilcoxon signed-ranks tests.

## Results

Our study was conducted on 112 patients, who were operated for aortic valve replacement during the study period, and divided into 2 groups. Fifty-two patients were operated through inverted T-shaped mini-sternotomy (MS); sixty-two patients were operated through a full median sternotomy (FS).

### Demographic data

The baseline characteristics of our sample are mentioned in Tables 1 and 2. There was no significant difference between both groups regarding the preoperative patients’ characteristics including age, gender, hypertension, hypercholesterolemia, diabetes mellitus, creatinine clearance, chronic obstructive pulmonary disease, preoperative renal dialysis, extra-cardiac arteriopathy, preoperative stroke, New York Heart Association (NYHA) class, preoperative EF%, preoperative angina, recent infarction, old infarction, urgency of the operation, preoperative EuroSCORE, valve lesion, valve pathology, preoperative atrial fibrillation, preoperative complete heart block, and preoperative hemoglobin level.

**Table 1:** Baseline clinical characteristics for the studied sample

		Mini-sternotomy (MS) (*n* = 50)		Full sternotomy (FS) (*n* = 52)		*p*
Age (years)	(Mean ± SD)^a^	41.1 ± 10.3		46.4 ± 10.3		0.72
	Min, max	28, 62		25, 71		
Gender	Male	37	(74)	33	(63.5)	0.29
Weight (kg)	(Mean ± SD)	78.1 ± 20.4		89.9 ± 13.5		0.02
Height (cm)	(Mean ± SD)	171.2 ± 10.3		171.4 ± 8.2		0.49
BMI (kg/m^2^)	(Mean ± SD)	26.98 ± 6.7		31.1 ± 5.7		0.001
BSA	(Mean ± SD)	1.88 ± 0.24		2.02 ± 1.7		0.001
Angina status	CCS 1	18	(36.0)	7	(13.5)	< 0.001
	CCS 2	24	(48.0)	11	(21.2)	
	CCS 3	8	(16.0)	33	(63.5)	
	CCS 4	0	(0.0)	1	(1.9)	
Dyspnea status	NYHA 1	5	(10)	0	(0)	
	NYHA 2	28	(56)	8	(15.4)	< 0.001
	NYHA 3	17	(34)	42	(80.8)	
	NYHA 4	0	(0)	2	(3.8)	
EuroSCORE %	(Mean ± SD)	1.99 ± 1.43		2.05 ± 1.77		0.64
Congestive cardiac failure	Never	50	(100)	50	(96.2)	0.49
	Past	0	(0)	2	(3.8)	
Diabetes mellitus^b^	Diet control	0	(0)	1	(1.9)	0.76
	Oral therapy	3	(6)	3	(5.8)	
	Insulin	2	(4)	3	(5.8)	
Hypertension		11	(22)	18	(34.6)	0.21
Hypothyroidism		0	(0)	1	(1.9)	0.24
Dyslipidemia		2	(4)	7	(13.5)	0.14
Smoking	Ex-smoker	9	(18)	14	(26.9)	0.53
	Active smoker	4	(8)	3	(5.7)	
GI tract	Liver dysfunction	1	(2)	1	(1.9)	1
Renal	Cr > 200 umol/l	0	(0)	3	(5.8)	0.24
Respiratory	Asthma	1	(2)	0	(0)	0.49
	COPD	1	(2)	1	(1.9)	
CVD	Stroke	1	(2)	1	(1.9)	1
PVD	Arteriopathy	1	(2)	2	(3.8)	1
Preoperative arrhythmia	Atrial fibrillation (AF)	1	(2)	1	(1.9)	1
Hemoglobin	(Mean ± SD)	13.5 ± 1.37		13.24 ± 1.79		0.35
	Min, max	10.2, 16.2		9.8, 17		
S. creatinine (mg/dl)	(Mean ± SD)	0.98 ± 0.39		0.84 ± 0.39		0.09
	Min, max	0.5, 3		0.2, 14		
Creatinine clearance (ml/min)	(Mean ± SD)	69.2 ± 22.63		77.72 ± 35.69		0.37
S. bilirubin	(Mean ± SD)	0.74 ± 0.37		0.48 ± 0.18		0.13
Coronary angiography		12	(24)	42	(80.8)	< 0.001

*BMI* body mass index; *COPD* chronic obstructive pulmonary disease; *MI* myocardial infarction; *PVD* peripheral vascular disease (or extracardiac arteriopathy) as indicated by claudication, amputation for arterial insufficiency, aortoiliac occlusive disease reconstruction, peripheral vascular bypass surgery, angioplasty or stent, or documented abdominal aortic aneurysm; *EuroSCORE* European System for Cardiac Operative Risk Evaluation; *CVD* cerebrovascular disease; *PVD* peripheral vascular disease or extracardiac arteriopathy

^a^The values are the number of patients with the percentage in brackets or the mean ± standard deviation or the median and interquartile range in brackets

^b^Basal serum glucose > 200 mg/dl at three consecutive measurements before surgery

**Table 2:** Echocardiographic data

		Mini-sternotomy (MS) (*n* = 50)		Full sternotomy (FS) (*n* = 52)		*p*
LVEF (%)	Fair (30–49%)^#^	5	(10)	7	(13.5)	0.58
	Good (> 50%)	45	(90)	45	(86.5)	
Aortic stenosis	*n* (%)	36	(72)	45	(86.5)	0.32
Aortic valve area (cm^2^)	(Mean ± SD)	1.24 ± 0.37		1.15 ± 0.53		0.72
Preoperative peak Gr. on the valve (mmHg)	(Mean ± SD)	71 ± 19.7		74.7 ± 23.7		0.38
Preoperative mean Gr. on the valve (mmHg)	(Mean ± SD)	43 ± 12.5		47 ± 17.9		0.68
Reason for operation	Severe valve pathology	22	(44)	0	(0)	< 0.001
	Anatomical	25	(50)	46	(88.5)	
	Angina	2	(4)	5	(9.6)	
	Unknown	1	(2)	1	(1.9)	

*LVEF* left ventricular ejection fraction, *Gr* gradient, *mmHg* millimeters of mercury

^#^Cases with LVEF < 40% were excluded as per exclusion criteria

**Fig. 3:**
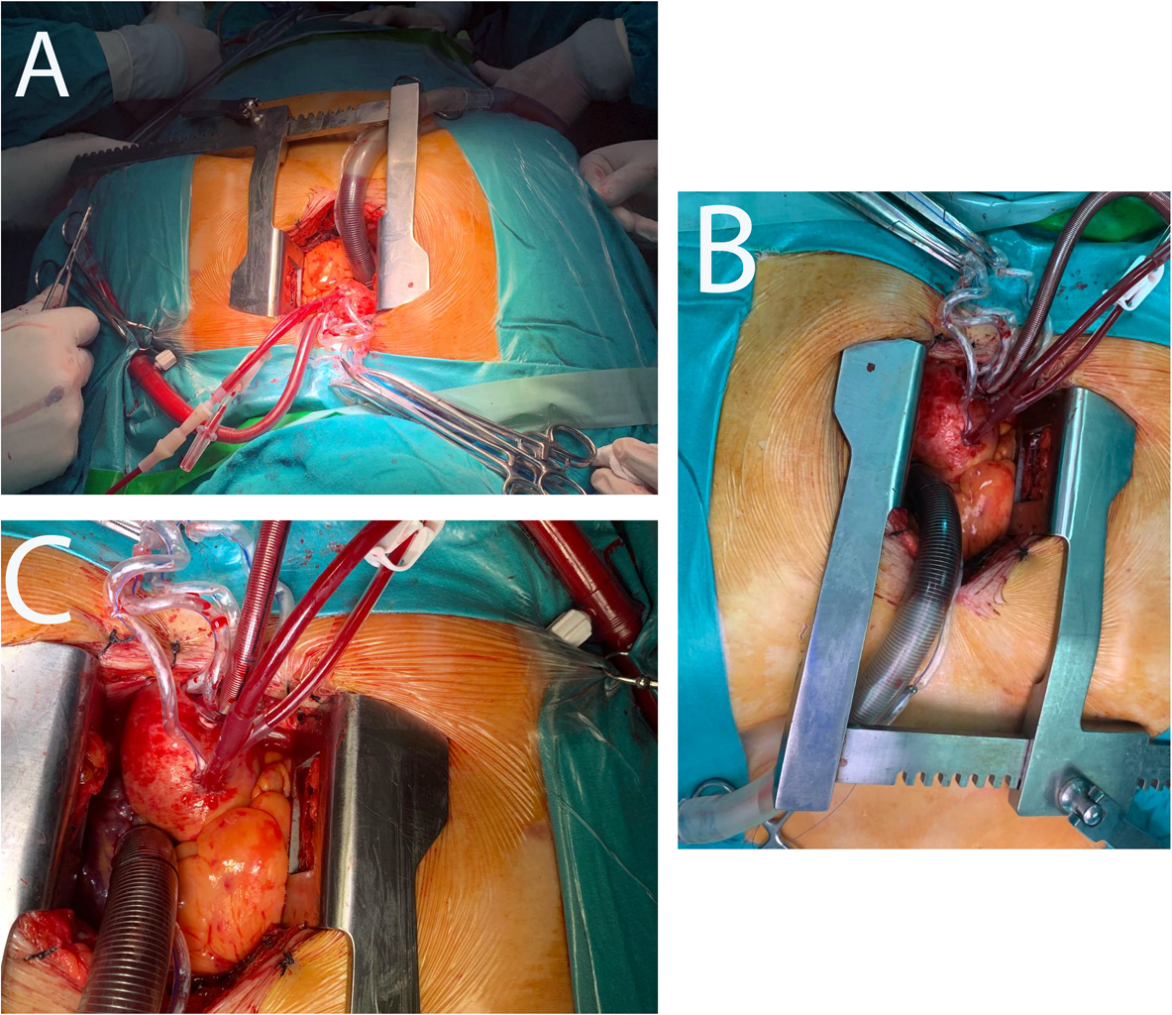
Exposure for minimally invasive aortic valve replacement in a morbidly obese female patient of BMI=60.2 kg/m^2^

Patients who underwent MIAVR had less body mass index (BMI) than the FS group (*p* = 0.002 and 0.001, respectively). We included a 54-year-old female morbidly obese diabetic and a hypertensive patient who had a BMI of 60.2 kg/m^2^ (obesity class III) who underwent isolate AVR using MS (Fig. 3).

### Operative and postoperative outcomes

All patients were operated for aortic valve replacement. Operative outcomes are mentioned in Table 3.

**Table 3:** Operative data for the studied sample

		Mini-sternotomy (MS) (*n* = 50)		Full sternotomy (FS) (*n* = 52)		*p*
Length of skin incision (cm)	(Mean ± SD)	5.82 ± 0.67		27.64 ± 2.71		0.001
Implant type	Mechanical	38	(76)	28	(53.8)	0.16
	Biological	12	(24)	24	(46.2)	
Implant size (mm)	19	4	(8)	9	(17.3)	0.31
	21	20	(40)	23	(44.2)	
	23	16	(32)	16	(30.8)	
	25	8	(16)	3	(5.8)	
	27	2	(4)	1	(0.2)	
Cumulative bypass time (min)	(Mean ± SD)	91.87 ± 34.41		94.91 ± 33.96		0.66
	Min, max	43, 180		50, 215		
Cumulative cross-clamp time (min)	Mean ± SD	68.88 ± 29.63		65.78 ± 24.36		0.58
	Min, max	70.2, 35		30, 165		
Minimum core temperature (°C)	Mean ± SD	31.02 ± 2.39		30.47 ± 1.17		0.14
	Min, max	31, 27		27, 32		
Inotropic support	High	0	(0)	3	(5.7)	0.445
	Moderate	1	(2)	2	(3.8)	
	Minimal	24	(48)	26	(50)	
	None	25	(50)	21	(38.7)	

*Cm* centimeters, *mm* millimeters, *SD* standard deviation, *°C* degree celcius

MS exhibited a significantly less skin incision length of 5.82 ± 0.67 cm versus 27.52 ± 2.71 cm (*p* = 0.001). No significant differences were observed as regards CCT and CPB times among both groups (*p* = 0.58 and 0.66).

Patients were monitored postoperatively as the standard of care. Postoperative complications are compared in Table 4. In the FS group, 68.3% had severe postoperative pain, compared with MS group, where 92% experienced mild pain at the visual analog scale at 12 postoperative hours. MS significantly reduced MV time (6.18 ± 1.86 versus 10.68 ± 12.78 h, *p* = 0.035), ICU stay (33.27 ± 19.75 versus 49.42 ± 47.1 h, *p* = 0.037), and blood transfusions (1.18 ± 0.89 versus 1.7 ± 0.97 units, *p* = 0.002). MS patients exhibited fewer pulmonary complications (pneumonia, reintubation, and pleural effusions) (*p* < 0.001), less superficial wound infections (*p* = 0.048), and no deep sternotomy wound infection (DSWI).

**Table 4:** Postoperative data

		Mini-sternotomy (MS) (*n* = 50)		Full sternotomy (FS) (*n* = 52)		*p* (95% CI)
**ICU and hospital stay**						
Ventilation time (days)	(Mean ± SD)	1 ± 0.1		1.17 ± 0.83		0.188 (− 0.417, 0.831)
	Min, max	1, 1		1, 6		
Ventilation time (h)	(Mean ± SD)	6.18 ± 1.86		10.68 ± 12.78		0.029 (− 6.63, − 1.09)
	Min, max	6, 18		4, 90		
ICU stay (nights)	(Mean ± SD)	1.43 ± 0.89		2.1 ± 1.89		0.035 (− 1.29, − 0.492)
	Min, max	1, 6		4, 10		
ICU stay (h)	(Mean ± SD)	33.27 ± 19.75		49.42 ± 47.1		0.037 (− 2.659, − 0.978)
	Min, max	18, 120		4, 240		
Postoperative hospital stay (days)	(Mean ± SD)	6.95 ± 3.83		8.34 ± 4.61		0.105 (− 3.156, 0.297)
	Min, max	2, 25		4, 132		
Postoperative complications		5	(10)	7	(11.3)	0.096
Transfusion number of blood units	(Mean ± SD)	1.18 ± 0.89		1.7 ± 0.97		0.002 (− 0.078, − 0.244)
	Min, max	0, 4		0, 5		
Need for transfusion (cases)	*n* (%)	34	(68)	38	(73.1)	0.052
Resternotomy	Bleeding/tamponade	2	(4)	5	(9.6)	0.437
Sternal resuturing		1	(2)	3	(5.8)	0.627
Arrhythmia	Ventricular fibrillation	0	(0)	3	(5.8)	0.014
	Supraventricular tachycardia	0	(0)	0	(0)	
	Permanent block—pace	2	(4)	1	(0.2)	
Pulmonary complications	Pleural effusion and drainage	1	(2)	2	(3.8)	< 0.001
	Reintubation/ventilation	0	(0)	4	(7.7)	
Neurological complications	Delayed recovery/stroke/other	0	(0)	1	(1.9)	0.138
	TIA	0	(0)	1	(1.9)	
Infective complications	Superficial wound infection	1	(2)	5	(9.6)	0.048 (− 0.194, − 0.179)
	DSWI	0	(0)	1	(1.9)	
Renal complications	Cr > 3	1	(2)	3	(5.7)	0.26
GI complications	Upper GI bleeding	0	(0)	4	(7.7)	0.22
Multi-organ failure	*n* (%)	0	(0)	2	(3.8)	0.34
Discharged to	Died in hospital	2	(4)	3	(5.7)	0.67
	Home	48	(96)	49	(94.2)	
Status at 2 years of follow-up	Alive	47	(94)	49	(94.2)	0.55
	Died in hospital	2	(4)	3	(5.7)	
	Died	1	(2)	0	(0)	

Resternotomy was indicated for bleeding in 7 patients. MS group showed less incidence of postoperative hemopericardium but without statistical significance. Four patients required sternal rewiring. A total all-cause operative mortality of 5 cases was reported (4.46%).

MS group had 2 mortalities; the first was a 58-year-old female diagnosed with severe aortic stenosis complicated with congestive heart failure (LVEF 42%). She was admitted to ICU on moderate inotropic support with low cardiac output, and mortality occurred on the second postoperative day. The second case was a 48-year-old male with aortic stenosis who was discharged to the ward following the third postoperative day with a stable condition, and mortality was encountered on the eighth postoperative day.

FS group had 3 mortality cases; the first was a 71-year-old male with severe aortic stenosis and LVEF of 40%. Low cardiac output and LVEF of 32% were encountered, and mortality occurred on the eighth postoperative day. The second case was a 65-year-old obese male smoker who was diagnosed with severe aortic regurgitation and impaired LVEF of 42%. Mortality was recorded following prolonged mechanical ventilation because of chest infection and sepsis. The third case was a 48-year-old nonsmoker diabetic female who had delayed recovery requiring reintubation and mechanical ventilation. She developed right-sided pneumonia. Sputum culture yielded Klebsiella species. She developed ventricular fibrillation for which she received cardioversion. Mortality was declared on the eighth postoperative day.

One late mortality was recorded upon follow-up of a 58-year-old female with a good left ventricular function who underwent SAVR using the MS approach 1 year following surgery due to cardiac arrest at home without prior symptoms. None of the mortality cases required reexploration or encountered bleeding.

### Cost analysis

All costs were converted to the corresponding US dollar rate using the inpatient hospital service Consumer Price Index (CPI). The total cost for admission and operation was calculated to discharge (direct cost). Indirect costs for sick leaves were not calculated.

Looking at the breakdown of costs (Table 5), significant cost reduction was recorded favoring MS approach as regards blood product cost (17.75%, *p* < 0.0001), ICU stay (34.48%, *p* < 0.0001), ward admission (15.46%, *p* < 0.0001), and total cost reduction of $580 (9.35%, *p* < 0.0001).

**Table 5:** Mean total cost breakdown

	Mini-sternotomy (MS) (*n* = 50)	Full sternotomy (FS) (*n* = 52)	Difference mean (%) (95% CI)	*p*
Operative costs				
Theater admission	$72.58	$72.58		NS
Oxygenator and tubing	$362.89	$362.89		NS
Cannulae (aortic and two stage venous)	$181.44	$181.44		NS
Drainage system	$38.72	$38.72		NS
Suturing material	$211.67	$211.67		NS
Sternal wire	$60.42	$90.42		NS
Mechanical prosthesis	$604.78	$604.78		NS
Biological prosthesis	$1632.95	$1632.95		NS
Blood	$241.92	$294.32	$52.08 (17.75%) (33.33, 70.83)	< 0.0001
Operating team fees	$2419.2	$2419.2		NS
Postoperative costs				
ICU stay and pharmacy	$529.77	$809.75	$279.23 (34.48%) (223.86, 334.61)	< 0.0001
Ventilator	$78.64	$78.64		NS
Ward	$349.6	$413.32	$63.72 (15.46%) (35.47, 91.98)	< 0.0001
Imaging	$54.25	$62.54	$8.29 (13.25%) (-3.975, 20.56)	0.1832
Laboratory	$415.75	$498.13	$82.38 (16.57%) (54.08, 110.69)	< 0.0001
Total cost (mechanical)	$5621.63	$6201.76	580.13 (9.35%) (351.08, 649.18)	< 0.0001
Total cost (biological)	$6649.81	$7229.93	580.12 (8.02%) (281.61, 719.38)	0.0001

*NS* non-significant statistically at *p* > 0.05

## Discussion

The lack of stable supplies is the foremost issue limiting us from performing minimally invasive cardiac surgery. Supplies may not be existing at all, or out of stock. As we are a government-funded institution, our supply chain is affected by the assets of the country, which are currently in a poor state.

In order to conquer some of these problems, we have had to espouse groundbreaking stratagems in other resource-poor settings to make our patients’ profit splendor of minimally invasive cardiac surgery. Minimal invasive surgery offers benefits more than being only cosmetically appealing. It has comparable results to the conventional FS [1].

Our study was novel in that it utilized itemized facility costs for the procedure for each technique. Our facility divided costs based on direct patient care (including blood, laboratory, theater, room, supplies, therapy, imaging, and miscellaneous costs).

It is clear from multiple studies [3] that antegrade perfusion through the ascending aorta has several advantages as being more physiological and reduces the risk of embolization, iatrogenic aortic dissection, and groin incisions with associated complications. We used the advantage of antegrade perfusion along the minimally invasive SAVR.

A recent Cochrane review [6] encompassed seven trials with 511 participants from 7 countries. The effects of minimally invasive limited upper MS on SAVR as compared with FS were investigated [7–13].

In our study, we found that CBP and CCT times were not significantly different between both techniques. Notably, total operative time was significantly longer in the MS group in our first cases. In accordance with our results, there was no evidence of an increase CBP with AVR performed via an upper MS (mean difference (MD) 3.02 min, 95% CI − 4.10, 10.14; participants = 311; studies = 5; low quality). There was no evidence of an increase in aortic CCT (MD 0.95 min, 95% CI − 3.45, 5.35; participants = 391; studies = 6; low quality) [7, 9–11, 13].

Unlike our study, none of the included studies in this review reported major adverse cardiac and cerebrovascular events as a composite endpoint. There was no evidence of any effect of upper MS on mortality versus FS (risk ratio (RR) 1.01, 95% (CI) 0.36 to 2.82; participants = 511; studies = 7; moderate quality) [9, 12].

We calculated the duration of postoperative MV from the moment of intensive care unit admission to the moment of extubation of the endotracheal tube. We observed a shorter period of postoperative MV in the MS group compared with the FS group. We contemplate that these results were due to the upheld integrity of the thoracic cage, expressly the lower costal margin, which helps in the conservation of the respiratory mechanics. The smaller incision can be convoyed with less pain and easier respiratory movements.

The results of mini-sternotomy are promising regarding decreasing MV and ICU stay, which will have a positive economic impact. Upper inverted T-shaped hemi-sternotomy maintains the integrity of the chest wall, which will improve the chest wall mechanics and facilitate early weaning and ambulation. This is a major advantage of MS over FS other than the cosmetic results and should be the primary target of the approach. In paradox, literature reported no significant effect on MV times (MD − 1.12 h, 95% CI − 3.43 to 1.19; participants = 297; studies = 5; low quality) [7, 8, 10–13].

We observed significantly shorter ICU stay a slightly shorter, but non-significant, duration of the postoperative hospital stay in the MS group. In the literature, there was a small reduction in length of intensive care unit stays as a result of the MS (MD was − 0.57 days, 95% CI − 0.93 to − 0.20; participants = 297; studies = 5; low quality) [9]. However, there was no evidence of an effect on length of hospital stay (MD − 1.31 days, 95% CI − 2.63, 0.01; participants = 297; studies = 5; I2 = 89%; very low quality) [7, 8, 10, 11 13].

In agreement to our results, postoperative blood loss was lower in MS (MD − 158.00 ml, 95% CI − 303.24 to − 12.76; participants = 297;studies = 5; moderate quality). The literature did not attest a drop in DSWI (RR 0.71, 95% CI 0.22 to 2.30; participants = 511; studies = 7; moderate quality) or re-exploration (risk ratio (RR) 1.01, 95% CI 0.48 to 2.13; participants = 511; studies = 7; moderate quality) [5, 6].

Dissimilar to our results, a review reported no change in pain scores by upper MS (standardized mean difference (SMD) − 0.33, 95% CI −0.85 to 0.20; participants = 197; studies = 3; I2 = 70%; very low quality) [8, 10, 11].

Our study did not address pulmonary function test as an endpoint. The literature reported a small increase in postoperative pulmonary function tests with MS (MD 1.98% predicted FEV1, 95% CI 0.62 to 3.33; participants = 257; studies = 4; I2 = 28%; low quality) [6].

MS approach did not reduce perioperative atrial fibrillation (AF) compared with FS (RR 0.60, 95% CI 0.07 to 4.89; participants = 240; studies = 3; moderate quality). All of our patients, except one, were having sinus rhythm [6].

None of the included studies in the Cochrane review reported cost analyses. This was a major advantage of the present study.

More recently, the MAVRIC (manubrium-limited mini-sternotomy versus conventional sternotomy for aortic valve replacement trial) [14] a single-center, single-blind randomized study that compared AVR via manubrium-limited mini-sternotomy using a 5- to 7-cm midline incision (intervention) and conventional FS and had postoperative red cell transfusion as the primary outcome. MIS was allied to higher CBP time and reduced drain losses, but this difference did not translate in a significant reduction in blood transfusion. Additionally, conventional SAVR was found to be more cost-effective (MS had a 5.8% probability of being cost-effective) [14].

### Study limitations and strength

There are some imperative shortcomings of this study. The prospective cohort study is non-randomized. The sample size was small, and the calculation was based on a single outcome of the ICU stay. Other vital outcomes were mentioned as secondary outcomes. Despite the orientation of all surgical teams with both approaches, several surgeons still preferred a traditional FS, so there might have been some selection bias as allocation was according to surgeons’ preference. The unequal number of groups was another issue. However, the groups were almost matched demographically. Despite the FS group had of higher BMI, we included cases with morbid obesity in the mini-sternotomy group up to 158 kg/BMI 60.2 kg/m^2^ without impact on postoperative outcomes. The study might be underpowered to detect significance in the difference in low-frequency events. This may or may not have affected some of the important outcomes such as pulmonary or neurological complications but not the ICU stay. Although we incorporated fast recovery protocols in our ICU, the duration of endotracheal intubation and length of postoperative stay was decided by the individual surgeon. No conversions were recorded.

Aortic surgery via mini-sternotomy has already been done in many centers worldwide, this study introduced MS at our centers with limited resources and achieved excellent outcomes with some benefits.

Evidently, the benefit of MS over FS is still debated, and some outcomes of each are well established as cosmetic superiority, bleeding reduction, postoperative pain, MV, and ICU stays as well as reduced pulmonary complications. In light of low resources, patients in developing countries still should benefit the splendor of MIAVR that has verified reduced budgets.

## Conclusions

With the lack of logistics and equipment in developing countries, MIAVR carries a significant reduction of blood product use, respiratory problems, pain, resource utilization, and cosmesis over FS without additional equipment. MIAVR using MS is feasible, with a rapid learning curve, in developing centers.

## Data Availability

The datasets used and/or analyzed during the current study available from the corresponding author on reasonable request.

## Abbreviations

BMI: Body mass index
CCT: Cumulative cross-clamp time
CI: Confidence interval
CPB: Cardiopulmonary bypass time
CPI: Consumer price index
DSWI: Deep sternotomy wound infection
EF%: Ejection fraction percentage
FS: Full sternotomy
ICU: Intensive care unit
MACEs: Major adverse cardiac events
MAVRIC: Manubrium-limited mini-sternotomy versus conventional sternotomy for aortic valve replacement trial
MIAVR: Minimally-invasive approaches to aortic valve surgery
MS: Mini-sternotomy/hemi-sternotomy
MV: Mechanical ventilation
NYHA: New York Heart Association class
RAT: Right anterior thoracotomy
RR: Risk ratio
SAVR: Surgical aortic valve replacement
SD: Standard deviation
SMD/MD: Standardized mean difference
